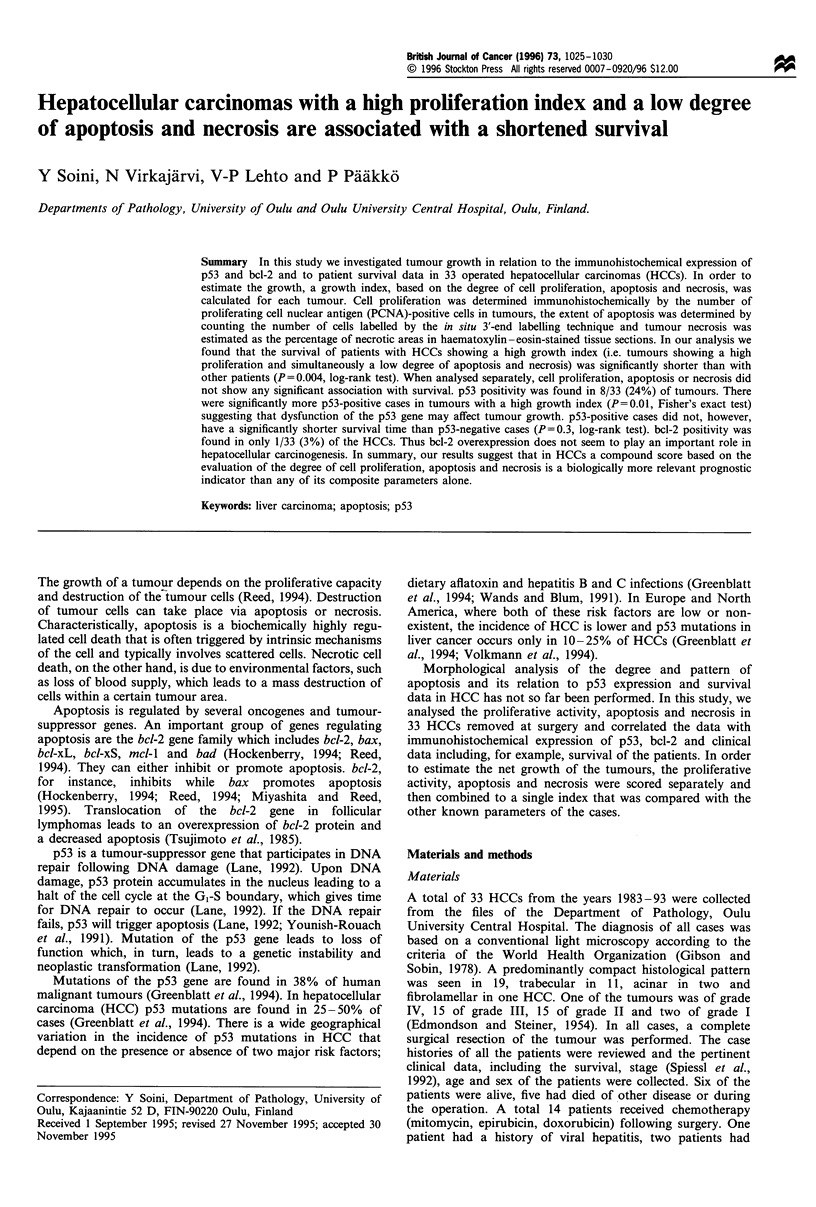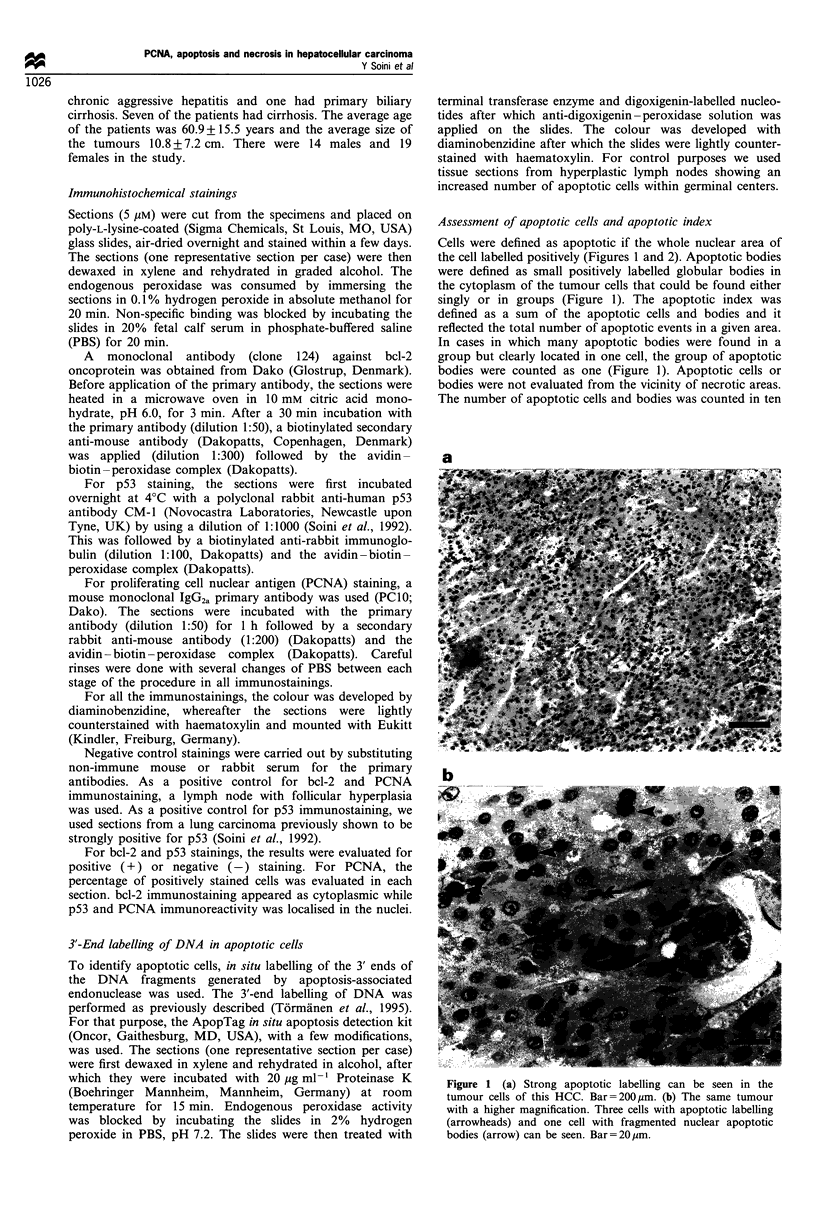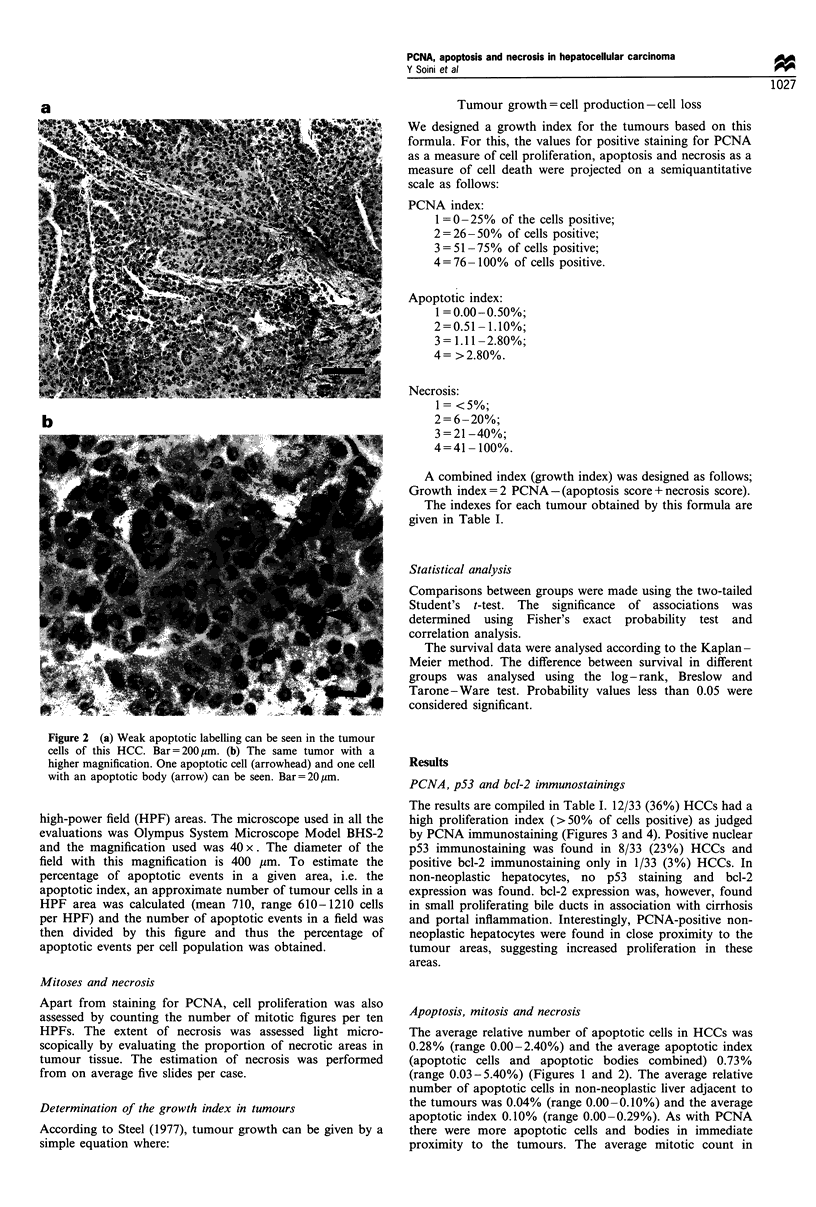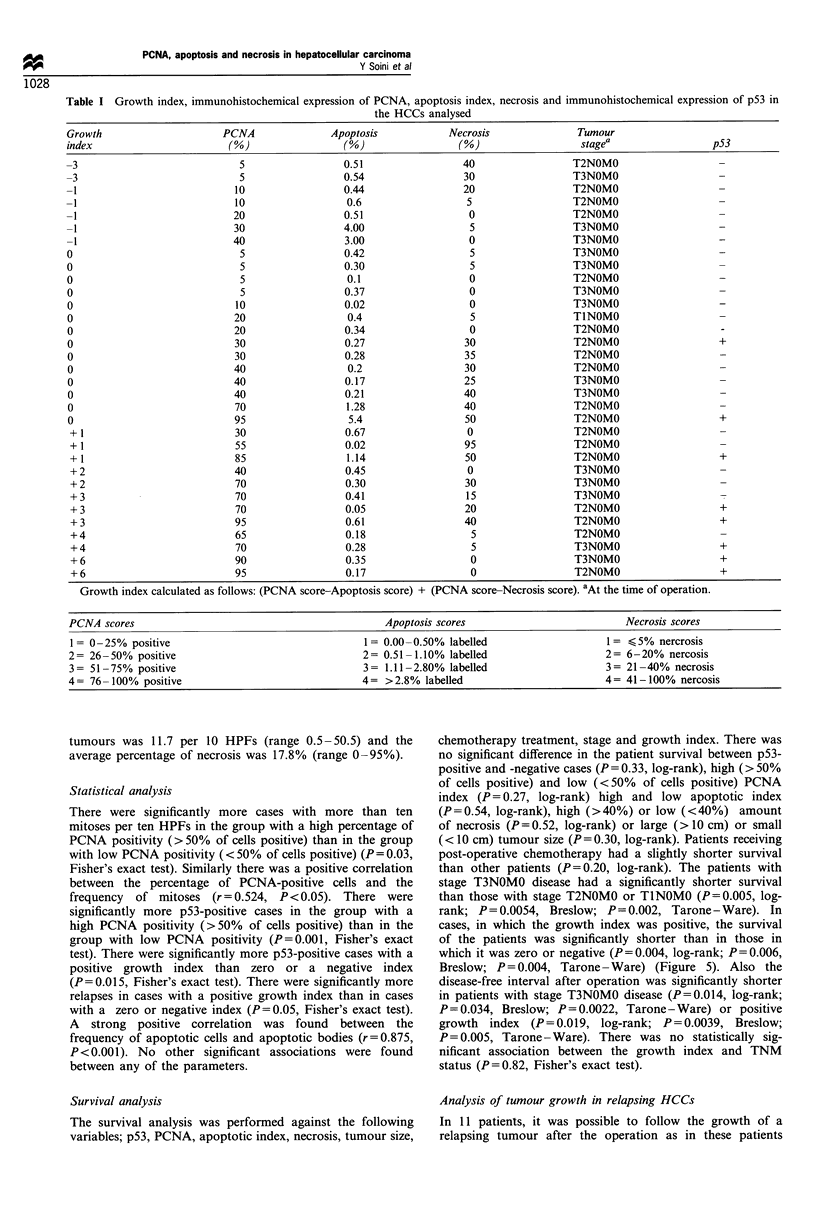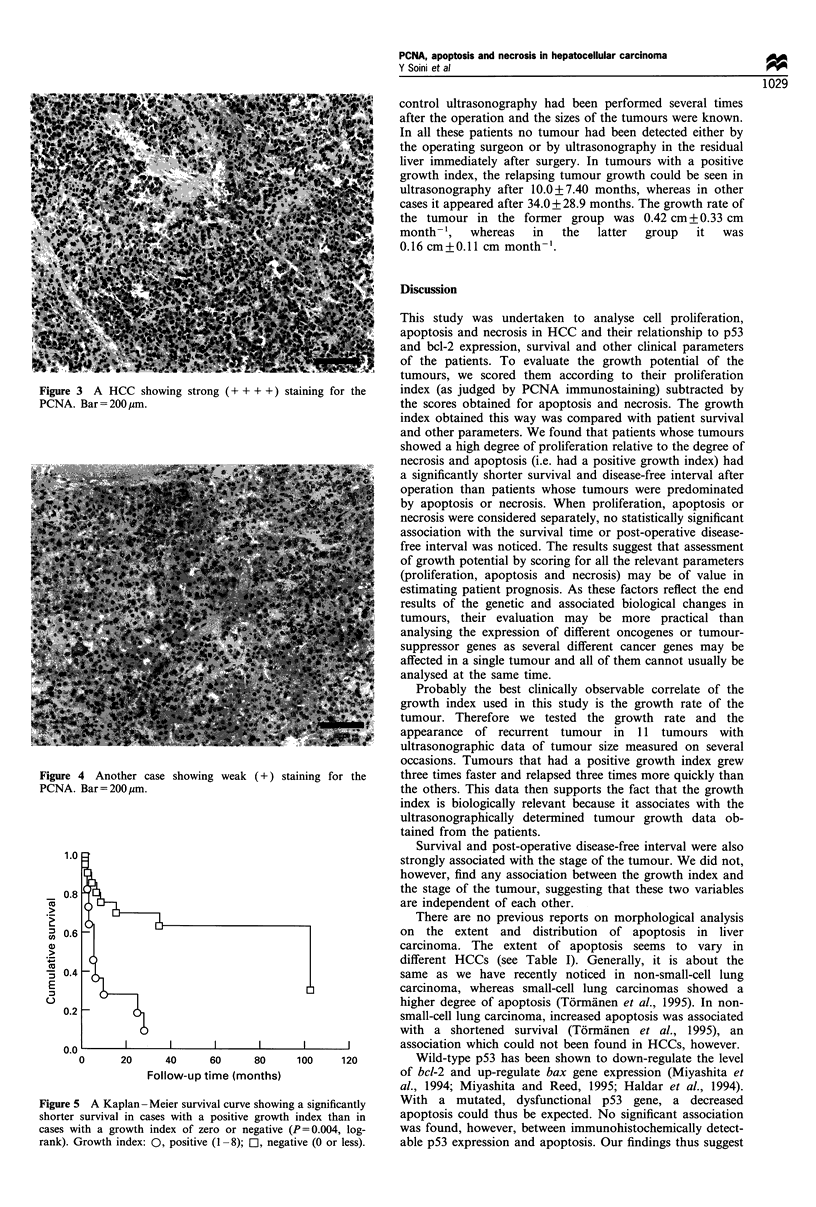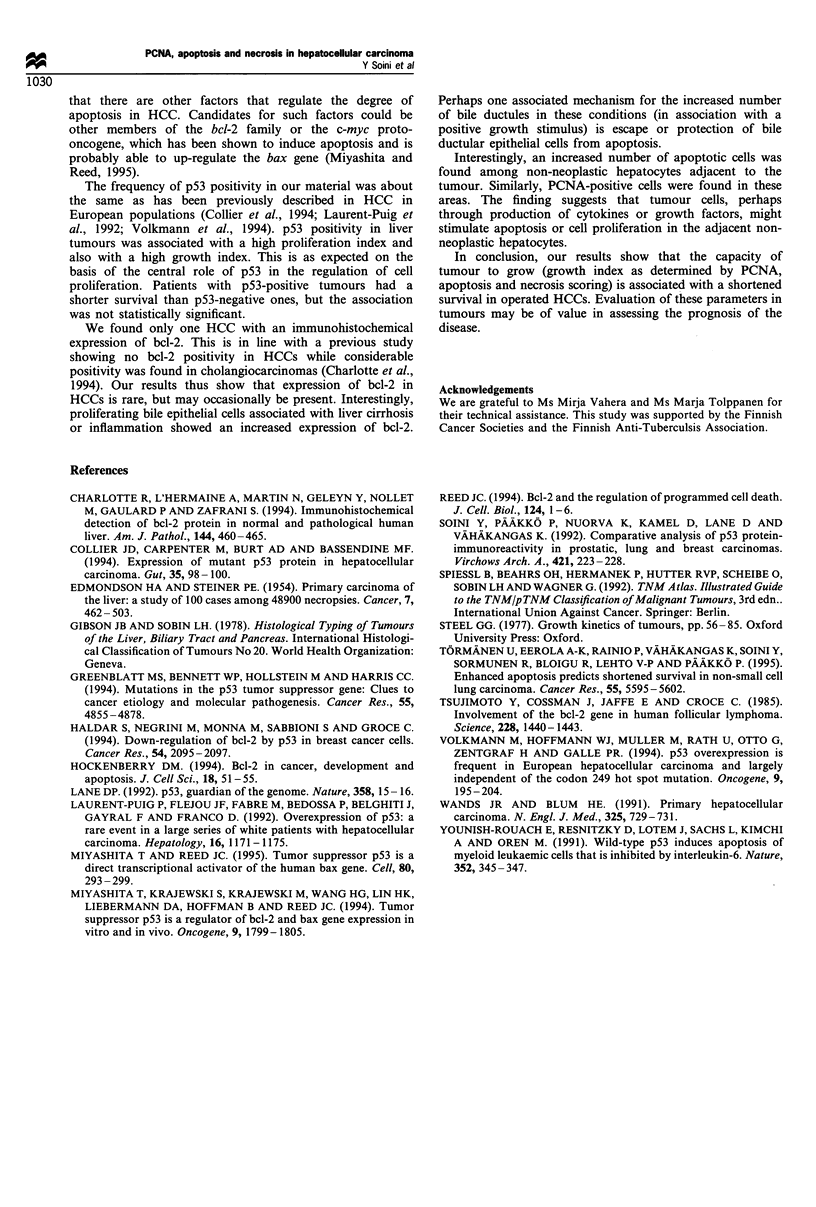# Hepatocellular carcinomas with a high proliferation index and a low degree of apoptosis and necrosis are associated with a shortened survival.

**DOI:** 10.1038/bjc.1996.199

**Published:** 1996-05

**Authors:** Y. Soini, N. Virkajärvi, V. P. Lehto, P. Pääkkö

**Affiliations:** Department of Pathology, University of Oulu, Finland.

## Abstract

**Images:**


British Journal of Cancer (1996) 73, 1025-1030
© 1996 Stockton Press All rights reserved 0007-0920/96 $12.00

### Hepatocellular carcinomas with a high proliferation index and a low degree of apoptosis and necrosis are associated with a shortened survival

Y Soini, N Virkajärvi, V-P Lehto and P Pääkkö

*Departments of Pathology, University of Oulu and Oulu University Central Hospital, Oulu, Finland.*

**Summary** In this study we investigated tumour growth in relation to the immunohistochemical expression of p53 and bcl-2 and to patient survival data in 33 operated hepatocellular carcinomas (HCCs). In order to estimate the growth, a growth index, based on the degree of cell proliferation, apoptosis and necrosis, was calculated for each tumour. Cell proliferation was determined immunohistochemically by the number of proliferating cell nuclear antigen (PCNA)-positive cells in tumours, the extent of apoptosis was determined by counting the number of cells labelled by the *in situ* 3′-end labelling technique and tumour necrosis was estimated as the percentage of necrotic areas in haematoxylin-eosin-stained tissue sections. In our analysis we found that the survival of patients with HCCs showing a high growth index (i.e. tumours showing a high proliferation and simultaneously a low degree of apoptosis and necrosis) was significantly shorter than with other patients ($P=0.004$, log-rank test). When analysed separately, cell proliferation, apoptosis or necrosis did not show any significant association with survival. p53 positivity was found in 8/33 (24%) of tumours. There were significantly more p53-positive cases in tumours with a high growth index ($P=0.01$, Fisher's exact test) suggesting that dysfunction of the p53 gene may affect tumour growth. p53-positive cases did not, however, have a significantly shorter survival time than p53-negative cases ($P=0.3$, log-rank test). bcl-2 positivity was found in only 1/33 (3%) of the HCCs. Thus bcl-2 overexpression does not seem to play an important role in hepatocellular carcinogenesis. In summary, our results suggest that in HCCs a compound score based on the evaluation of the degree of cell proliferation, apoptosis and necrosis is a biologically more relevant prognostic indicator than any of its composite parameters alone.

**Keywords:** liver carcinoma; apoptosis; p53

The growth of a tumour depends on the proliferative capacity and destruction of the tumour cells (Reed, 1994). Destruction of tumour cells can take place via apoptosis or necrosis. Characteristically, apoptosis is a biochemically highly regulated cell death that is often triggered by intrinsic mechanisms of the cell and typically involves scattered cells. Necrotic cell death, on the other hand, is due to environmental factors, such as loss of blood supply, which leads to a mass destruction of cells within a certain tumour area.

Apoptosis is regulated by several oncogenes and tumour-suppressor genes. An important group of genes regulating apoptosis are the *bcl*-2 gene family which includes *bcl*-2, *bax*, *bcl*-xL, *bcl*-xS, *mcl*-1 and *bad* (Hockenberry, 1994; Reed, 1994). They can either inhibit or promote apoptosis. *bcl*-2, for instance, inhibits while *bax* promotes apoptosis (Hockenberry, 1994; Reed, 1994; Miyashita and Reed, 1995). Translocation of the *bcl*-2 gene in follicular lymphomas leads to an overexpression of *bcl*-2 protein and a decreased apoptosis (Tsujimoto *et al.*, 1985).

p53 is a tumour-suppressor gene that participates in DNA repair following DNA damage (Lane, 1992). Upon DNA damage, p53 protein accumulates in the nucleus leading to a halt of the cell cycle at the $G_1$-S boundary, which gives time for DNA repair to occur (Lane, 1992). If the DNA repair fails, p53 will trigger apoptosis (Lane, 1992; Younish-Rouach *et al.*, 1991). Mutation of the p53 gene leads to loss of function which, in turn, leads to a genetic instability and neoplastic transformation (Lane, 1992).

Mutations of the p53 gene are found in 38% of human malignant tumours (Greenblatt *et al.*, 1994). In hepatocellular carcinoma (HCC) p53 mutations are found in 25-50% of cases (Greenblatt *et al.*, 1994). There is a wide geographical variation in the incidence of p53 mutations in HCC that depend on the presence or absence of two major risk factors; dietary aflatoxin and hepatitis B and C infections (Greenblatt *et al.*, 1994; Wands and Blum, 1991). In Europe and North America, where both of these risk factors are low or non-existent, the incidence of HCC is lower and p53 mutations in liver cancer occurs only in 10-25% of HCCs (Greenblatt *et al.*, 1994; Volkmann *et al.*, 1994).

Morphological analysis of the degree and pattern of apoptosis and its relation to p53 expression and survival data in HCC has not so far been performed. In this study, we analysed the proliferative activity, apoptosis and necrosis in 33 HCCs removed at surgery and correlated the data with immunohistochemical expression of p53, bcl-2 and clinical data including, for example, survival of the patients. In order to estimate the net growth of the tumours, the proliferative activity, apoptosis and necrosis were scored separately and then combined to a single index that was compared with the other known parameters of the cases.

### Materials and methods

### *Materials*

A total of 33 HCCs from the years 1983-93 were collected from the files of the Department of Pathology, Oulu University Central Hospital. The diagnosis of all cases was based on a conventional light microscopy according to the criteria of the World Health Organization (Gibson and Sobin, 1978). A predominantly compact histological pattern was seen in 19, trabecular in 11, acinar in two and fibrolamellar in one HCC. One of the tumours was of grade IV, 15 of grade III, 15 of grade II and two of grade I (Edmondson and Steiner, 1954). In all cases, a complete surgical resection of the tumour was performed. The case histories of all the patients were reviewed and the pertinent clinical data, including the survival, stage (Spiessl *et al.*, 1992), age and sex of the patients were collected. Six of the patients were alive, five had died of other disease or during the operation. A total 14 patients received chemotherapy (mitomycin, epirubicin, doxorubicin) following surgery. One patient had a history of viral hepatitis, two patients had

Correspondence: Y Soini, Department of Pathology, University of Oulu, Kajaanintie 52 D, FIN-90220 Oulu, Finland
Received 1 September 1995; revised 27 November 1995; accepted 30 November 1995

chronic aggressive hepatitis and one had primary biliary cirrhosis. Seven of the patients had cirrhosis. The average age of the patients was 60.9 ± 15.5 years and the average size of the tumours 10.8 ± 7.2 cm. There were 14 males and 19 females in the study.

### *Immunohistochemical stainings*

Sections (5 $\mu$M) were cut from the specimens and placed on poly-L-lysine-coated (Sigma Chemicals, St Louis, MO, USA) glass slides, air-dried overnight and stained within a few days. The sections (one representative section per case) were then dewaxed in xylene and rehydrated in graded alcohol. The endogenous peroxidase was consumed by immersing the sections in 0.1% hydrogen peroxide in absolute methanol for 20 min. Non-specific binding was blocked by incubating the slides in 20% fetal calf serum in phosphate-buffered saline (PBS) for 20 min.

A monoclonal antibody (clone 124) against bcl-2 oncoprotein was obtained from Dako (Glostrup, Denmark). Before application of the primary antibody, the sections were heated in a microwave oven in 10 mM citric acid monohydrate, pH 6.0, for 3 min. After a 30 min incubation with the primary antibody (dilution 1:50), a biotinylated secondary anti-mouse antibody (Dakopatts, Copenhagen, Denmark) was applied (dilution 1:300) followed by the avidin–biotin–peroxidase complex (Dakopatts).

For p53 staining, the sections were first incubated overnight at 4°C with a polyclonal rabbit anti-human p53 antibody CM-1 (Novocastra Laboratories, Newcastle upon Tyne, UK) by using a dilution of 1:1000 (Soini *et al*., 1992). This was followed by a biotinylated anti-rabbit immunoglobulin (dilution 1:100, Dakopatts) and the avidin–biotin–peroxidase complex (Dakopatts).

For proliferating cell nuclear antigen (PCNA) staining, a mouse monoclonal $IgG_{2a}$ primary antibody was used (PC10; Dako). The sections were incubated with the primary antibody (dilution 1:50) for 1 h followed by a secondary rabbit anti-mouse antibody (1:200) (Dakopatts) and the avidin–biotin–peroxidase complex (Dakopatts). Careful rinses were done with several changes of PBS between each stage of the procedure in all immunostainings.

For all the immunostainings, the colour was developed by diaminobenzidine, whereafter the sections were lightly counterstained with haematoxylin and mounted with Eukitt (Kindler, Freiburg, Germany).

Negative control stainings were carried out by substituting non-immune mouse or rabbit serum for the primary antibodies. As a positive control for bcl-2 and PCNA immunostaining, a lymph node with follicular hyperplasia was used. As a positive control for p53 immunostaining, we used sections from a lung carcinoma previously shown to be strongly positive for p53 (Soini *et al*., 1992).

For bcl-2 and p53 stainings, the results were evaluated for positive (+) or negative (−) staining. For PCNA, the percentage of positively stained cells was evaluated in each section. bcl-2 immunostaining appeared as cytoplasmic while p53 and PCNA immunoreactivity was localised in the nuclei.

### *3′-End labelling of DNA in apoptotic cells*

To identify apoptotic cells, *in situ* labelling of the 3′ ends of the DNA fragments generated by apoptosis-associated endonuclease was used. The 3′-end labelling of DNA was performed as previously described (Törmänen *et al*., 1995). For that purpose, the ApopTag *in situ* apoptosis detection kit (Oncor, Gaithesburg, MD, USA), with a few modifications, was used. The sections (one representative section per case) were first dewaxed in xylene and rehydrated in alcohol, after which they were incubated with 20 $\mu$g ml$^{-1}$ Proteinase K (Boehringer Mannheim, Mannheim, Germany) at room temperature for 15 min. Endogenous peroxidase activity was blocked by incubating the slides in 2% hydrogen peroxide in PBS, pH 7.2. The slides were then treated with terminal transferase enzyme and digoxigenin-labelled nucleotides after which anti-digoxigenin–peroxidase solution was applied on the slides. The colour was developed with diaminobenzidine after which the slides were lightly counterstained with haematoxylin. For control purposes we used tissue sections from hyperplastic lymph nodes showing an increased number of apoptotic cells within germinal centers.

### *Assessment of apoptotic cells and apoptotic index*

Cells were defined as apoptotic if the whole nuclear area of the cell labelled positively (Figures 1 and 2). Apoptotic bodies were defined as small positively labelled globular bodies in the cytoplasm of the tumour cells that could be found either singly or in groups (Figure 1). The apoptotic index was defined as a sum of the apoptotic cells and bodies and it reflected the total number of apoptotic events in a given area. In cases in which many apoptotic bodies were found in a group but clearly located in one cell, the group of apoptotic bodies were counted as one (Figure 1). Apoptotic cells or bodies were not evaluated from the vicinity of necrotic areas. The number of apoptotic cells and bodies was counted in ten

**Figure 1** (**a**) Strong apoptotic labelling can be seen in the tumour cells of this HCC. Bar = 200 $\mu$m. (**b**) The same tumour with a higher magnification. Three cells with apoptotic labelling (arrowheads) and one cell with fragmented nuclear apoptotic bodies (arrow) can be seen. Bar = 20 $\mu$m.

**Figure 2** (**a**) Weak apoptotic labelling can be seen in the tumour cells of this HCC. Bar = 200 $\mu$m. (**b**) The same tumor with a higher magnification. One apoptotic cell (arrowhead) and one cell with an apoptotic body (arrow) can be seen. Bar = 20 $\mu$m.

high-power field (HPF) areas. The microscope used in all the evaluations was Olympus System Microscope Model BHS-2 and the magnification used was 40×. The diameter of the field with this magnification is 400 $\mu$m. To estimate the percentage of apoptotic events in a given area, i.e. the apoptotic index, an approximate number of tumour cells in a HPF area was calculated (mean 710, range 610–1210 cells per HPF) and the number of apoptotic events in a field was then divided by this figure and thus the percentage of apoptotic events per cell population was obtained.

*Mitoses and necrosis*

Apart from staining for PCNA, cell proliferation was also assessed by counting the number of mitotic figures per ten HPFs. The extent of necrosis was assessed light microscopically by evaluating the proportion of necrotic areas in tumour tissue. The estimation of necrosis was performed from on average five slides per case.

*Determination of the growth index in tumours*

According to Steel (1977), tumour growth can be given by a simple equation where:

Tumour growth = cell production − cell loss

We designed a growth index for the tumours based on this formula. For this, the values for positive staining for PCNA as a measure of cell proliferation, apoptosis and necrosis as a measure of cell death were projected on a semiquantitative scale as follows:

PCNA index:
1 = 0–25% of the cells positive;
2 = 26–50% of cells positive;
3 = 51–75% of cells positive;
4 = 76–100% of cells positive.

Apoptotic index:
1 = 0.00–0.50%;
2 = 0.51–1.10%;
3 = 1.11–2.80%;
4 = >2.80%.

Necrosis:

1 = <5%;

2 = 6–20%;

3 = 21–40%;

4 = 41–100%.

A combined index (growth index) was designed as follows; Growth index = 2 PCNA − (apoptosis score + necrosis score).

The indexes for each tumour obtained by this formula are given in Table I.

*Statistical analysis*

Comparisons between groups were made using the two-tailed Student's *t*-test. The significance of associations was determined using Fisher's exact probability test and correlation analysis.

The survival data were analysed according to the Kaplan–Meier method. The difference between survival in different groups was analysed using the log–rank, Breslow and Tarone–Ware test. Probability values less than 0.05 were considered significant.

### Results

*PCNA, p53 and bcl-2 immunostainings*

The results are compiled in Table I. 12/33 (36%) HCCs had a high proliferation index (>50% of cells positive) as judged by PCNA immunostaining (Figures 3 and 4). Positive nuclear p53 immunostaining was found in 8/33 (23%) HCCs and positive bcl-2 immunostaining only in 1/33 (3%) HCCs. In non-neoplastic hepatocytes, no p53 staining and bcl-2 expression was found. bcl-2 expression was, however, found in small proliferating bile ducts in association with cirrhosis and portal inflammation. Interestingly, PCNA-positive non-neoplastic hepatocytes were found in close proximity to the tumour areas, suggesting increased proliferation in these areas.

*Apoptosis, mitosis and necrosis*

The average relative number of apoptotic cells in HCCs was 0.28% (range 0.00–2.40%) and the average apoptotic index (apoptotic cells and apoptotic bodies combined) 0.73% (range 0.03–5.40%) (Figures 1 and 2). The average relative number of apoptotic cells in non-neoplastic liver adjacent to the tumours was 0.04% (range 0.00–0.10%) and the average apoptotic index 0.10% (range 0.00–0.29%). As with PCNA there were more apoptotic cells and bodies in immediate proximity to the tumours. The average mitotic count in

**Table I** Growth index, immunohistochemical expression of PCNA, apoptosis index, necrosis and immunohistochemical expression of p53 in the HCCs analysed

| *Growth index* | *PCNA (%)* | *Apoptosis (%)* | *Necrosis (%)* | *Tumour stage*[a] | *p53* |
|---|---|---|---|---|---|
| −3 | 5 | 0.51 | 40 | T2N0M0 | − |
| −3 | 5 | 0.54 | 30 | T3N0M0 | − |
| −1 | 10 | 0.44 | 20 | T2N0M0 | − |
| −1 | 10 | 0.6 | 5 | T2N0M0 | − |
| −1 | 20 | 0.51 | 0 | T2N0M0 | − |
| −1 | 30 | 4.00 | 5 | T3N0M0 | − |
| −1 | 40 | 3.00 | 0 | T3N0M0 | − |
| 0 | 5 | 0.42 | 5 | T3N0M0 | − |
| 0 | 5 | 0.30 | 5 | T3N0M0 | − |
| 0 | 5 | 0.1 | 0 | T2N0M0 | − |
| 0 | 5 | 0.37 | 0 | T3N0M0 | − |
| 0 | 10 | 0.02 | 0 | T3N0M0 | − |
| 0 | 20 | 0.4 | 5 | T1N0M0 | − |
| 0 | 20 | 0.34 | 0 | T2N0M0 | - |
| 0 | 30 | 0.27 | 30 | T2N0M0 | + |
| 0 | 30 | 0.28 | 35 | T2N0M0 | − |
| 0 | 40 | 0.2 | 30 | T2N0M0 | − |
| 0 | 40 | 0.17 | 25 | T3N0M0 | − |
| 0 | 40 | 0.21 | 40 | T3N0M0 | − |
| 0 | 70 | 1.28 | 40 | T2N0M0 | − |
| 0 | 95 | 5.4 | 50 | T2N0M0 | + |
| +1 | 30 | 0.67 | 0 | T2N0M0 | − |
| +1 | 55 | 0.02 | 95 | T2N0M0 | − |
| +1 | 85 | 1.14 | 50 | T2N0M0 | + |
| +2 | 40 | 0.45 | 0 | T3N0M0 | − |
| +2 | 70 | 0.30 | 30 | T3N0M0 | − |
| +3 | 70 | 0.41 | 15 | T3N0M0 | − |
| +3 | 70 | 0.05 | 20 | T2N0M0 | + |
| +3 | 95 | 0.61 | 40 | T2N0M0 | + |
| +4 | 65 | 0.18 | 5 | T2N0M0 | − |
| +4 | 70 | 0.28 | 5 | T3N0M0 | + |
| +6 | 90 | 0.35 | 0 | T3N0M0 | + |
| +6 | 95 | 0.17 | 0 | T2N0M0 | + |

Growth index calculated as follows: (PCNA score–Apoptosis score) + (PCNA score–Necrosis score). [a]At the time of operation.

| *PCNA scores* | *Apoptosis scores* | *Necrosis scores* |
|---|---|---|
| 1= 0–25% positive | 1= 0.00–0.50% labelled | 1= ≤5% nercrosis |
| 2= 26–50% positive | 2= 0.51–1.10% labelled | 2= 6–20% nercosis |
| 3= 51–75% positive | 3= 1.11–2.80% labelled | 3= 21–40% necrosis |
| 4= 76–100% positive | 4= >2.8% labelled | 4= 41–100% nercosis |

tumours was 11.7 per 10 HPFs (range 0.5–50.5) and the average percentage of necrosis was 17.8% (range 0–95%).

### *Statistical analysis*

There were significantly more cases with more than ten mitoses per ten HPFs in the group with a high percentage of PCNA positivity (>50% of cells positive) than in the group with low PCNA positivity (<50% of cells positive) ($P=0.03$, Fisher's exact test). Similarly there was a positive correlation between the percentage of PCNA-positive cells and the frequency of mitoses ($r=0.524$, $P<0.05$). There were significantly more p53-positive cases in the group with a high PCNA positivity (>50% of cells positive) than in the group with low PCNA positivity ($P=0.001$, Fisher's exact test). There were significantly more p53-positive cases with a positive growth index than zero or a negative index ($P=0.015$, Fisher's exact test). There were significantly more relapses in cases with a positive growth index than in cases with a zero or negative index ($P=0.05$, Fisher's exact test). A strong positive correlation was found between the frequency of apoptotic cells and apoptotic bodies ($r=0.875$, $P<0.001$). No other significant associations were found between any of the parameters.

### *Survival analysis*

The survival analysis was performed against the following variables; p53, PCNA, apoptotic index, necrosis, tumour size, chemotherapy treatment, stage and growth index. There was no significant difference in the patient survival between p53-positive and -negative cases ($P=0.33$, log-rank), high (>50% of cells positive) and low (<50% of cells positive) PCNA index ($P=0.27$, log-rank) high and low apoptotic index ($P=0.54$, log-rank), high (>40%) or low (<40%) amount of necrosis ($P=0.52$, log-rank) or large (>10 cm) or small (<10 cm) tumour size ($P=0.30$, log-rank). Patients receiving post-operative chemotherapy had a slightly shorter survival than other patients ($P=0.20$, log-rank). The patients with stage T3N0M0 disease had a significantly shorter survival than those with stage T2N0M0 or T1N0M0 ($P=0.005$, log-rank; $P=0.0054$, Breslow; $P=0.002$, Tarone–Ware). In cases, in which the growth index was positive, the survival of the patients was significantly shorter than in those in which it was zero or negative ($P=0.004$, log-rank; $P=0.006$, Breslow; $P=0.004$, Tarone–Ware) (Figure 5). Also the disease-free interval after operation was significantly shorter in patients with stage T3N0M0 disease ($P=0.014$, log-rank; $P=0.034$, Breslow; $P=0.0022$, Tarone–Ware) or positive growth index ($P=0.019$, log-rank; $P=0.0039$, Breslow; $P=0.005$, Tarone–Ware). There was no statistically significant association between the growth index and TNM status ($P=0.82$, Fisher's exact test).

### *Analysis of tumour growth in relapsing HCCs*

In 11 patients, it was possible to follow the growth of a relapsing tumour after the operation as in these patients

**Figure 3** A HCC showing strong (+ + + +) staining for the PCNA. Bar = 200 μm.

**Figure 4** Another case showing weak (+) staining for the PCNA. Bar = 200 μm.

**Figure 5** A Kaplan–Meier survival curve showing a significantly shorter survival in cases with a positive growth index than in cases with a growth index of zero or negative ($P = 0.004$, log-rank). Growth index: ○, positive (1–8); □, negative (0 or less).

control ultrasonography had been performed several times after the operation and the sizes of the tumours were known. In all these patients no tumour had been detected either by the operating surgeon or by ultrasonography in the residual liver immediately after surgery. In tumours with a positive growth index, the relapsing tumour growth could be seen in ultrasonography after $10.0 \pm 7.40$ months, whereas in other cases it appeared after $34.0 \pm 28.9$ months. The growth rate of the tumour in the former group was $0.42$ cm $\pm 0.33$ cm month$^{-1}$, whereas in the latter group it was $0.16$ cm $\pm 0.11$ cm month$^{-1}$.

### Discussion

This study was undertaken to analyse cell proliferation, apoptosis and necrosis in HCC and their relationship to p53 and bcl-2 expression, survival and other clinical parameters of the patients. To evaluate the growth potential of the tumours, we scored them according to their proliferation index (as judged by PCNA immunostaining) subtracted by the scores obtained for apoptosis and necrosis. The growth index obtained this way was compared with patient survival and other parameters. We found that patients whose tumours showed a high degree of proliferation relative to the degree of necrosis and apoptosis (i.e. had a positive growth index) had a significantly shorter survival and disease-free interval after operation than patients whose tumours were predominated by apoptosis or necrosis. When proliferation, apoptosis or necrosis were considered separately, no statistically significant association with the survival time or post-operative disease-free interval was noticed. The results suggest that assessment of growth potential by scoring for all the relevant parameters (proliferation, apoptosis and necrosis) may be of value in estimating patient prognosis. As these factors reflect the end results of the genetic and associated biological changes in tumours, their evaluation may be more practical than analysing the expression of different oncogenes or tumour-suppressor genes as several different cancer genes may be affected in a single tumour and all of them cannot usually be analysed at the same time.

Probably the best clinically observable correlate of the growth index used in this study is the growth rate of the tumour. Therefore we tested the growth rate and the appearance of recurrent tumour in 11 tumours with ultrasonographic data of tumour size measured on several occasions. Tumours that had a positive growth index grew three times faster and relapsed three times more quickly than the others. This data then supports the fact that the growth index is biologically relevant because it associates with the ultrasonographically determined tumour growth data obtained from the patients.

Survival and post-operative disease-free interval were also strongly associated with the stage of the tumour. We did not, however, find any association between the growth index and the stage of the tumour, suggesting that these two variables are independent of each other.

There are no previous reports on morphological analysis on the extent and distribution of apoptosis in liver carcinoma. The extent of apoptosis seems to vary in different HCCs (see Table I). Generally, it is about the same as we have recently noticed in non-small-cell lung carcinoma, whereas small-cell lung carcinomas showed a higher degree of apoptosis (Törmänen *et al.*, 1995). In non-small-cell lung carcinoma, increased apoptosis was associated with a shortened survival (Törmänen *et al.*, 1995), an association which could not been found in HCCs, however.

Wild-type p53 has been shown to down-regulate the level of *bcl-2* and up-regulate *bax* gene expression (Miyashita *et al.*, 1994; Miyashita and Reed, 1995; Haldar *et al.*, 1994). With a mutated, dysfunctional p53 gene, a decreased apoptosis could thus be expected. No significant association was found, however, between immunohistochemically detectable p53 expression and apoptosis. Our findings thus suggest that there are other factors that regulate the degree of apoptosis in HCC. Candidates for such factors could be other members of the *bcl*-2 family or the c-*myc* proto-oncogene, which has been shown to induce apoptosis and is probably able to up-regulate the *bax* gene (Miyashita and Reed, 1995).

The frequency of p53 positivity in our material was about the same as has been previously described in HCC in European populations (Collier *et al.*, 1994; Laurent-Puig *et al.*, 1992; Volkmann *et al.*, 1994). p53 positivity in liver tumours was associated with a high proliferation index and also with a high growth index. This is as expected on the basis of the central role of p53 in the regulation of cell proliferation. Patients with p53-positive tumours had a shorter survival than p53-negative ones, but the association was not statistically significant.

We found only one HCC with an immunohistochemical expression of bcl-2. This is in line with a previous study showing no bcl-2 positivity in HCCs while considerable positivity was found in cholangiocarcinomas (Charlotte *et al.*, 1994). Our results thus show that expression of bcl-2 in HCCs is rare, but may occasionally be present. Interestingly, proliferating bile epithelial cells associated with liver cirrhosis or inflammation showed an increased expression of bcl-2. Perhaps one associated mechanism for the increased number of bile ductules in these conditions (in association with a positive growth stimulus) is escape or protection of bile ductular epithelial cells from apoptosis.

Interestingly, an increased number of apoptotic cells was found among non-neoplastic hepatocytes adjacent to the tumour. Similarly, PCNA-positive cells were found in these areas. The finding suggests that tumour cells, perhaps through production of cytokines or growth factors, might stimulate apoptosis or cell proliferation in the adjacent non-neoplastic hepatocytes.

In conclusion, our results show that the capacity of tumour to grow (growth index as determined by PCNA, apoptosis and necrosis scoring) is associated with a shortened survival in operated HCCs. Evaluation of these parameters in tumours may be of value in assessing the prognosis of the disease.

**Acknowledgements**
We are grateful to Ms Mirja Vahera and Ms Marja Tolppanen for their technical assistance. This study was supported by the Finnish Cancer Societies and the Finnish Anti-Tuberculsis Association.

### References

CHARLOTTE R, L'HERMAINE A, MARTIN N, GELEYN Y, NOLLET M, GAULARD P AND ZAFRANI S. (1994). Immunohistochemical detection of bcl-2 protein in normal and pathological human liver. *Am. J. Pathol.*, **144**, 460–465.

COLLIER JD, CARPENTER M, BURT AD AND BASSENDINE MF. (1994). Expression of mutant p53 protein in hepatocellular carcinoma. *Gut*, **35**, 98–100.

EDMONDSON HA AND STEINER PE. (1954). Primary carcinoma of the liver: a study of 100 cases among 48900 necropsies. *Cancer*, **7**, 462–503.

GIBSON JB AND SOBIN LH. (1978). *Histological Typing of Tumours of the Liver, Biliary Tract and Pancreas*. International Histological Classification of Tumours No 20. World Health Organization: Geneva.

GREENBLATT MS, BENNETT WP, HOLLSTEIN M AND HARRIS CC. (1994). Mutations in the p53 tumor suppressor gene: Clues to cancer etiology and molecular pathogenesis. *Cancer Res.*, **55**, 4855–4878.

HALDAR S, NEGRINI M, MONNA M, SABBIONI S AND GROCE C. (1994). Down-regulation of bcl-2 by p53 in breast cancer cells. *Cancer Res.*, **54**, 2095–2097.

HOCKENBERRY DM. (1994). Bcl-2 in cancer, development and apoptosis. *J. Cell Sci.*, **18**, 51–55.

LANE DP. (1992). p53, guardian of the genome. *Nature*, **358**, 15–16.

LAURENT-PUIG P, FLEJOU JF, FABRE M, BEDOSSA P, BELGHITI J, GAYRAL F AND FRANCO D. (1992). Overexpression of p53: a rare event in a large series of white patients with hepatocellular carcinoma. *Hepatology*, **16**, 1171–1175.

MIYASHITA T AND REED JC. (1995). Tumor suppressor p53 is a direct transcriptional activator of the human bax gene. *Cell*, **80**, 293–299.

MIYASHITA T, KRAJEWSKI S, KRAJEWSKI M, WANG HG, LIN HK, LIEBERMANN DA, HOFFMAN B AND REED JC. (1994). Tumor suppressor p53 is a regulator of bcl-2 and bax gene expression in vitro and in vivo. *Oncogene*, **9**, 1799–1805.

REED JC. (1994). Bcl-2 and the regulation of programmed cell death. *J. Cell. Biol.*, **124**, 1–6.

SOINI Y, PÄÄKKÖ P, NUORVA K, KAMEL D, LANE D AND VÄHÄKANGAS K. (1992). Comparative analysis of p53 protein-immunoreactivity in prostatic, lung and breast carcinomas. *Virchows Arch. A.*, **421**, 223–228.

SPIESSL B, BEAHRS OH, HERMANEK P, HUTTER RVP, SCHEIBE O, SOBIN LH AND WAGNER G. (1992). *TNM Atlas. Illustrated Guide to the TNM/pTNM Classification of Malignant Tumours*, 3rd edn.. International Union Against Cancer. Springer: Berlin.

STEEL GG. (1977). Growth kinetics of tumours, pp. 56–85. Oxford University Press: Oxford.

TÖRMÄNEN U, EEROLA A-K, RAINIO P, VÄHÄKANGAS K, SOINI Y, SORMUNEN R, BLOIGU R, LEHTO V-P AND PÄÄKKÖ P. (1995). Enhanced apoptosis predicts shortened survival in non-small cell lung carcinoma. *Cancer Res.*, **55**, 5595–5602.

TSUJIMOTO Y, COSSMAN J, JAFFE E AND CROCE C. (1985). Involvement of the bcl-2 gene in human follicular lymphoma. *Science*, **228**, 1440–1443.

VOLKMANN M, HOFFMANN WJ, MULLER M, RATH U, OTTO G, ZENTGRAF H AND GALLE PR. (1994). p53 overexpression is frequent in European hepatocellular carcinoma and largely independent of the codon 249 hot spot mutation. *Oncogene*, **9**, 195–204.

WANDS JR AND BLUM HE. (1991). Primary hepatocellular carcinoma. *N. Engl. J. Med.*, **325**, 729–731.

YOUNISH-ROUACH E, RESNITZKY D, LOTEM J, SACHS L, KIMCHI A AND OREN M. (1991). Wild-type p53 induces apoptosis of myeloid leukaemic cells that is inhibited by interleukin-6. *Nature*, **352**, 345–347.